# Comparative Transcriptome Analysis Reveals the Underlying Response Mechanism to Salt Stress in Maize Seedling Roots

**DOI:** 10.3390/metabo13111155

**Published:** 2023-11-16

**Authors:** Chen Zhang, Bin Chen, Ping Zhang, Qinghui Han, Guangwu Zhao, Fucheng Zhao

**Affiliations:** 1 College of Advanced Agricultural Science, Zhejiang Agriculture and Forestry University, Lin’an 311300, China; zchen@stu.zafu.edu.cn (C.Z.);; 2 Institute of Maize and Featured Upland Crops, Zhejiang Academy of Agricultural Sciences, Dongyang 322100, China; chenbin@zaas.ac.cn (B.C.);

**Keywords:** root system of maize seedlings, salt stress, RNA-Seq, differences in salt tolerance

## Abstract

Crop growth and development can be impeded by salt stress, leading to a significant decline in crop yield and quality. This investigation performed a comparative analysis of the physiological responses of two maize inbred lines, namely L318 (CML115) and L323 (GEMS58), under salt-stress conditions. The results elucidated that CML115 exhibited higher salt tolerance compared with GEMS58. Transcriptome analysis of the root system revealed that DEGs shared by the two inbred lines were significantly enriched in the MAPK signaling pathway–plant and plant hormone signal transduction, which wield an instrumental role in orchestrating the maize response to salt-induced stress. Furthermore, the DEGs’ exclusivity to salt-tolerant genotypes was associated with sugar metabolism pathways, and these unique DEGs may account for the disparities in salt tolerance between the two genotypes. Meanwhile, we investigated the dynamic global transcriptome in the root systems of seedlings at five time points after salt treatment and compared transcriptome data from different genotypes to examine the similarities and differences in salt tolerance mechanisms of different germplasms.

## 1. Introduction

Salt stress is a prevalent environmental factor that has a substantial impact on global crop production and hinders agricultural advancements [1,2,3,4]. The expansion of salinized farmland can be attributed to various factors, including climate conditions, terrain variations, inefficient irrigation, and the fertilization methods employed in agricultural practices [5,6]. Excessive soil salinity hampers the process of seed germination, root elongation, and seed implantation, ultimately resulting in a diminished crop yield and compromised quality [7]. In the case of salt-sensitive crops, high salt concentrations can even lead to crop mortality. Research on plant responses to salt stress has suggested that elevated salt levels disrupt the osmotic pressure in plants, hindering water absorption by roots. Simultaneously, the excessive accumulation of salt ions can cause ion toxicity, posing a significant threat to plant growth [8]. Consequently, the response mechanism of plants to high-salt environments plays a crucial role in determining their salt tolerance. This mechanism is usually achieved with the extent of root salt absorption [9,10], regulation of reactive oxygen species (ROS) levels [11], and regulation of salt-stress-related genes and transcription factors [12].

Maize (*Zea mays* L.) is a widely cultivated crop known for its high yield, ease of management, and versatile uses in agriculture and industry. Nevertheless, maize exhibits greater vulnerability to salt stress compared with other crops [13,14]. Recent research has identified specific genes and regulatory factors associated with salt stress in maize. One example is the type-A response regulator (*ZmRR1*), which can control the release of Cl^−^ from shoots and interacts with cytokinin to inhibit its function, thereby negatively regulating plant salt tolerance [15]. The maize AP2-ERF family member *ZmEREB20* positively regulates salt tolerance through the molecular mechanism associated with hormone signaling, ROS scavenging, and root hair plasticity. Its overexpressed lines have a higher survival rate under salt stress [16]. WRKYs are an important family of transcription factors that widely participate in plant development, defense regulation, and stress responses. The heterologous overexpression of *ZmWRKY114* in rice decreases salt-stress tolerance and sensitivity to ABA by regulating the expression of certain ABA signal pathways and stress-responsive genes [17].

Plant salt tolerance is a complex quantitative trait [18] that has been extensively investigated using various methodologies, including QTL mapping [19], genome-wide association studies [20], and RNA sequencing [21]. Transcriptome analysis is particularly valuable in understanding the response of plants to salt stress, identifying important genes and pathways associated with salt tolerance, and elucidating the mechanisms by which plants sense and respond to salt stress. Researchers analyzed the transcriptome data of maize at different time points after exposure to salt stress and found that the response of the transcriptome to salt stress was rapid and transient [22]. Through the analysis of gene expression patterns in salt-tolerant and salt-sensitive sorghum varieties, researchers found that secondary metabolism and hormone signaling pathways exhibited faster responses in the early stages of salt stress. Furthermore, salt-tolerant varieties displayed a greater efficacy in restoring plant homeostasis when compared with their salt-sensitive counterparts [23]. Another study focused on the transcriptome comparison between the salt tolerance of the parental lines of Maize Hybrid An’nong876, revealing the significant impacts of salt stress on the redox and photosynthesis pathways. It was also found that the regulation of transcription factors and hormone signal pathways played a crucial role in the response of maize to salt stress [24].

On the basis of comparing salt tolerance levels, this study selected two inbred maize lines, L323 (CML115) and L318 (GEMS58), for global transcriptome analysis, and the investigation led to the identification of two regulatory pathways responsible for salt tolerance in maize, along with the discovery of key genes associated with the salt-stress response. These findings offer significant genetic resources that can be utilized for the development of maize varieties with enhanced salt tolerance.

## 2. Materials and Methods

### 2.1. Plant Materials and Experimental Treatment

The seeds of L323 and L318 were sterilized using a solution of 75% alcohol and 5% NaClO prior to being planted in a substrate for seedlings. Once the plants reached the three-leaf and one-heart stage, the root substrate was thoroughly rinsed with distilled water and then all the plants were transferred to a 1/2 Hoagland nutrient solution for one day to acclimate to the hydroponic environment. Subsequently, the plants subjected to salt stress were grown in a 1/2 Hoagland nutrient solution supplemented with 150 mmol/L of NaCl, while the control group was grown in the same nutrient solution without NaCl. The seedlings were cultivated in plant growth chambers under conditions of 25/20 °C (16 h light/8 h dark) with a photosynthetic photon flux density of 350 μmol·m^−2^·s^−1^ and a relative humidity of 60%. The nutrient solution was replaced every two days during the experiment.

### 2.2. Measurements of Physiological Parameters

The root water content of the seedlings was calculated with the drying–weighing method. After measuring the fresh weight (W_f_) of the roots, the roots were deactivated at 105 °C for 15 min, then dried to a constant weight at 80 °C to measure the dry weight (W_d_). RWC = (W_f_ − W_d_)/W_f_ × 100%. The malondialdehyde (MDA) content, Peroxidase (POD) activity, and Catalase (CAT) activity were measured using an assay kit in accordance with the instructions provided by the manufacturer (Solarbio Science & Technology Co., Ltd., Beijing, China).

### 2.3. Sample Collection and Acquisition of Transcriptome Data

Maize seedling roots were collected for RNA sequencing at 6, 12, 24, and 48 h after being exposed to salt stress, using untreated seedlings as controls. Three biological replicates were set for each treatment, and three seedlings were selected for each replicate. Guangzhou Genedenovo Biotechnology Co., Ltd. (Guangzhou, China), conducted transcriptome sequencing and obtained gene expression data from 30 samples.

### 2.4. RNA Extraction and RNA-Seq

The total RNA was extracted using a Trizol reagent kit (Invitrogen, Carlsbad, CA, USA) following the manufacturer’s protocol. RNA quality was assessed on an Agilent 2100 Bioanalyzer (Agilent Technologies, Palo Alto, CA, USA) and checked using RNase-free agarose gel electrophoresis. After the total RNA was extracted, eukaryotic mRNA was enriched using Oligo (dT) beads. Then, the mRNA was fragmented using a fragmentation buffer and reversely transcribed into cDNA. The resulting cDNA library was sequenced using an Illumina Novaseq 6000. The raw reads were filtered to obtain high-quality clean reads by removing reads containing adapters, reads containing more than 10% unknown nucleotides (N), and low-quality reads containing more than 50% low-quality (*q*-value ≤ 20) bases. Differential expression analysis was performed with DESeq2_1.42.0 [25] software between two different groups (and with edgeR [26] between two samples). The genes/transcripts with the parameters of a false discovery rate (FDR) ≤ 0.05 and a |log_2_FC| ≥ 1 were considered DEGs.

### 2.5. Trend Analysis

To examine the expression pattern of DEGs, the expression data of each sample (in the order of treatment) were normalized to 0, log_2_ (v_1_/v_0_), log_2_ (v_2_/v_0_), and then clustered with Short Time-series Expression Miner software (STEM) version 1.3.13. The parameters were set as follows: profiles 20, ratio 1.0000 [log_2_2 = 1, log_2_1.5 = 0.5849625]. The clustered profiles with a *p*-value ≤ 0.05 were considered significant profiles.

### 2.6. GO and KEGG Enrichment Analysis

All DEGs were mapped to Gene Ontology (GO) terms using the Gene Ontology database (http://www.geneontology.org/ accessed on 20 June 2023). GO terms meeting an FDR ≤ 0.05 were defined as significantly enriched GO terms in DEGs. KEGG [27] is the primary public database for pathway-related information. Compared to the entire genome background, pathways meeting an FDR ≤ 0.05 were defined as significantly enriched pathways in DEGs.

### 2.7. Quantitative Real-Time PCR

To validate the accuracy of the Illumina sequencing results, seven DEGs were randomly selected for qRT-PCR. The total RNA was extracted from the same samples that were used for sequencing. The cDNA was synthesized using a HiScript III 1st Strand cDNA Synthesis Kit (+gDNA wiper) (Vazyme Biotech Co., Ltd., Nanjing, China). The primer sequences used were designed using a free online primer design tool (https://www.ncbi.nlm.nih.gov/ accessed on 17 August 2023) and synthesized by Tsingke Biotechnology (Beijing, China) Co., Ltd. The maize Cyanase gene was used as the reference gene. The primer sequences for qRT-PCR are listed in Table S6. qRT-PCR was performed on a QuantStudio™ 5 System (Thermo Fisher Scientific, Waltham, MA, USA) using a ChamQ Universal SYBR qPCR Master Mix (Vazyme Biotech Co., Ltd., Nanjing, China) following the provided instructions. The relative expression level of target genes was calculated using the 2^−ΔΔCT^ method [28].

### 2.8. Statistical Analysis

An analysis of variance (ANOVA) was performed using the IBMSPSS statistical package (version 27.0.1). The bar and line graphs were drawn with the Graphpad Prism 9.5 software program.

## 3. Results

### 3.1. Phenotypic and Physiological Responses of Two Inbred Maize Lines to Salt Stress

**Figure 1:**
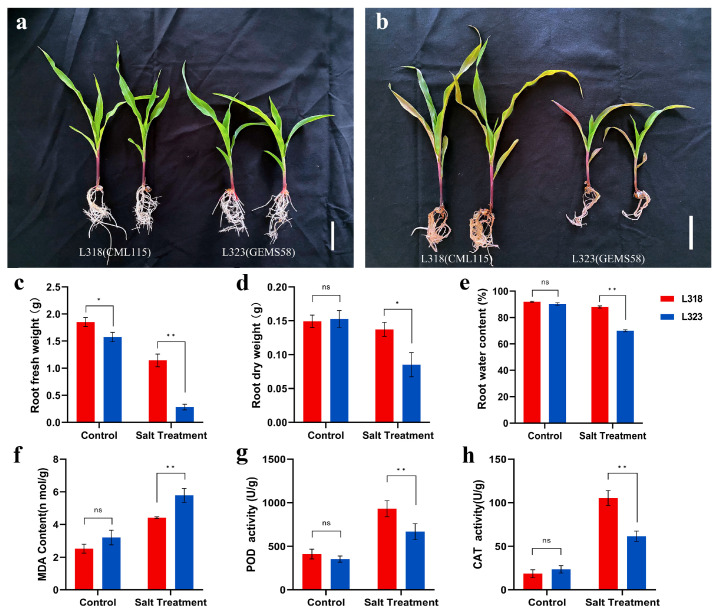
Phenotypic and physiological responses of CML115 and GEMS58 seedlings under control and salt-stress conditions. (**a**) Phenotypic responses of control seedlings after 7 days. (**b**) Phenotypic responses of seedlings after 7 days of salt treatment. Scale bar = 5 cm. (**c**–**e**) Root fresh weight, root dry weight, and root water content. (**f**–**h**) MDA content, POD activity, and CAT activity. Each bar chart represents the average ± SD of three biological replicates; ns, not significant; * *p* ≤ 0.05; ** *p* ≤ 0.01.

In order to assess the salt tolerance of inbred maize lines and the response of seedlings to salt stress, L323 and L318 seedlings at the V3 stage were cultivated in a 1/2 Hoagland nutrient solution. The control group was not exposed to NaCl, while the experimental group was subjected to a concentration of 150 mmol/L of NaCl for a duration of 7 days. The impact of salt stress on root system growth inhibition and leaf wilting was found to be more pronounced in L323 compared with L318, as observed through the examination of seedling and root morphology (Figure 1a,b). Salt stress resulted in a 7.9% reduction in plant dry matter in L318, while the reductions in L323 were significantly higher. Both fresh and dry weights decreased in the salt treatment of both plants when compared with the control (Figure 1c,d). The root water content in L318 was similar between the salt-stress (87.96%) and control groups (91.94%). However, in L323, it decreased under salt stress (69.95%) compared with the control (90.28%) (Figure 1e). Under controlled conditions, there were no differences in the levels of MDA content, POD activity, or CAT activity in the roots of the two seedling lines. However, under salt-stress conditions, the POD and CAT activities of the L318 root system were significantly higher than those of L323, while the MDA content was significantly lower in L318 compared with L323 (Figure 1f–h). Based on these findings, L318 was classified as a salt-tolerant inbred line, while L323 was classified as a salt-sensitive one.

### 3.2. Transcriptome Analysis

To investigate the characteristics of gene expression, sequencing libraries were prepared from the root system of maize seedlings that were subjected to different durations of NaCl treatment (0, 6, 12, 24, and 48 h). A total of thirty libraries were sequenced, consisting of five salt-stress durations, three biological replicates, and two inbred lines. To ensure the reliability of the data, low-quality reads were removed, resulting in a total of 1,287,158,622 clean reads from 30 libraries for comparative analysis. These clean reads were aligned to the *Zea mays* reference transcriptome Zm-B73-REFERENCE-GRAMENE-4.0, with an average mapping rate of 81.77% (Tables S1 and S2).

**Figure 2:**
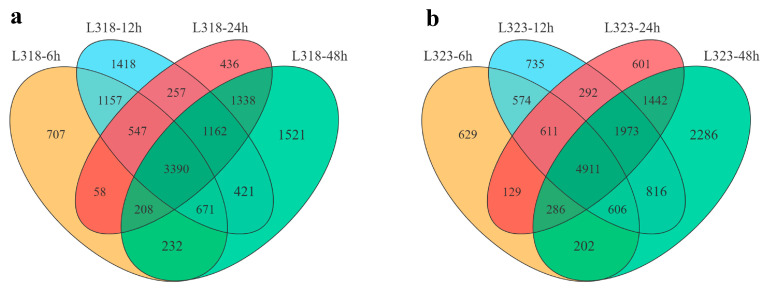
(**a**) A Venn diagram of the differentially expressed genes in the salt-tolerant line L318 during the four salt treatment stages. (**b**) A Venn diagram of differentially expressed genes of the salt-sensitive line L323 during the four salt treatment stages.

DESeq software was utilized to analyze all genes that were differentially expressed. DEGs were identified based on the criteria of |log_2_(FC)| ≥ 1 and an FDR ≤ 0.05. In comparison with the gene expression at 0 h, there were 6970 (6 h), 9023 (12 h), 7396 (24 h), and 8943 (48 h) DEGs in L318, respectively. Furthermore, 3390 DEGs remained consistently present throughout the entire duration of the salt treatment (Figure 2a). Similarly, there were 7948, 10,518, 10,254, and 12,522 DEGs in L323 after salt treatment for 6, 12, 24, and 48 h, respectively. Among these genes, 4911 displayed a differential expression across all time points (Figure 2b).

### 3.3. GO and KEGG Enrichment Analysis of DEGs Involved in Salt Tolerance Response

**Figure 3:**
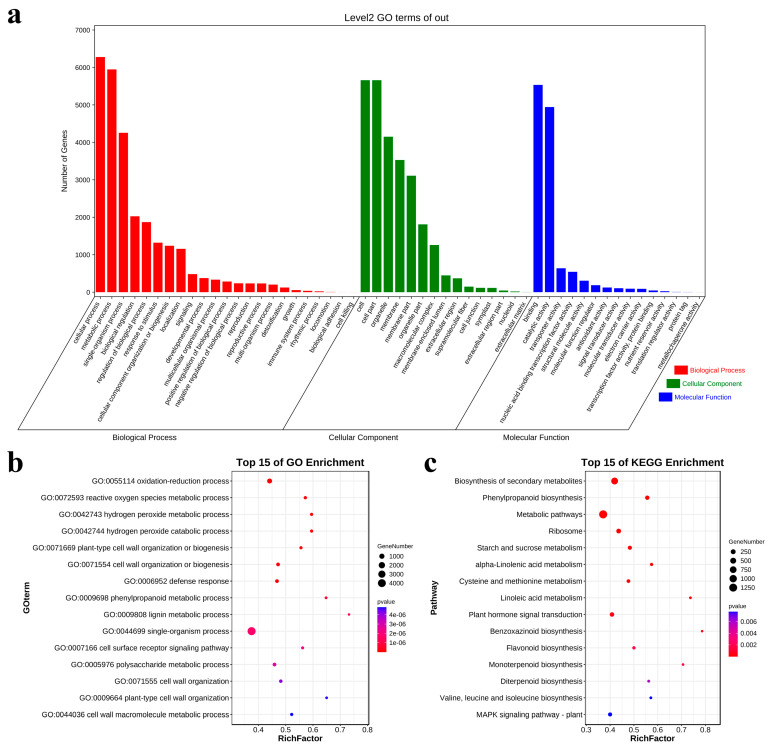
(**a**) The common DEGs of the two inbred lines were divided into three categories: biological process, molecular function, and cellular components with GO analysis. (**b**) Bubble plots of the top 15 GO items in the GO enrichment analysis of DEGs shared by two inbred lines. (**c**) Bubble plots of the top 15 KEGG items in the GO enrichment analysis of DEGs shared by two inbred lines.

To investigate the relationship between DEGs to salt stress and salt tolerance, we conducted a Gene Ontology (GO) enrichment analysis on DEGs that were shared by two inbred lines and the DEGs specific to the salt-tolerant line. The DEGs were classified into three functional groups: biological process (BP), cellular component (CC), and molecular function (MF). The analysis revealed that the differentially expressed genes in root transcripts were primarily associated with biological process functions, as shown in Figure 3a.

The bubble plot (Figure 3b) depicting the enrichment analysis of the Gene Ontology (GO) reveals the fifteen most prominent biological processes in which DEGs were found to be significantly enriched under conditions of salt stress. These genes were found to be enriched in pathways related to the catabolic metabolism of reactive oxygen species, including the oxidation-reduction process (GO:0055114), reactive oxygen species metabolic process (GO:0072593), hydrogen peroxide metabolic process (GO:0042743), and hydrogen peroxide catabolic process (GO:0042744). Additionally, they were enriched in defense response (GO:0006952), the phenylpropanoid metabolic process (GO:0009698), the cell surface receptor signaling pathway (GO:0007166), the secondary metabolic process (GO:0019748), the phenylpropanoid catabolic process (GO:0046271), and the other metabolic processes related to plant stress resistance. Meanwhile, several biological processes related to plant cell walls were also enriched, such as plant-type cell wall organization or biogenesis (GO:0071669), cell wall organization or biogenesis (GO:0071554), cell wall organization (GO:0071555), plant-type cell wall organization (GO:0009664), the cell wall macromolecule metabolic process (GO:0044036), and the cell wall polysaccharide metabolic process (GO:0010383).

To further reveal the functions and roles of these DEGs in salt-stress response, a KEGG enrichment analysis was performed on these DEGs. These common DEGs were enriched in a total of 30 pathways, including the biosynthesis of the secondary metabolite (ko01110); phenylpropanoid biosynthesis (ko00940); metabolic pathways (ko01100); ribosomes (ko03010); starch and sucrose metabolism (ko00500); alpha-linolenic acid metabolism (ko00592); cysteine and methionine metabolism (ko00270); linoleic acid metabolism (ko00591); plant hormone signal transduction (ko04075); benzoxazinoid biosynthesis (ko00402); flavonoid biosynthesis (ko00941); diterpenoid biosynthesis (ko00904); valine, leucine, and isoleucine biosynthesis (ko00290); and the MAPK signaling pathway–plant (ko04016). The biosynthesis of secondary metabolites and metabolic pathways were enriched with the highest number of DEGs, with 796 and 1316 DEGs, respectively. In addition, the plant hormone signal transduction and MAPK signaling pathway–plant were enriched in 173 and 116 DEGs (Figure 3c).

**Figure 4:**
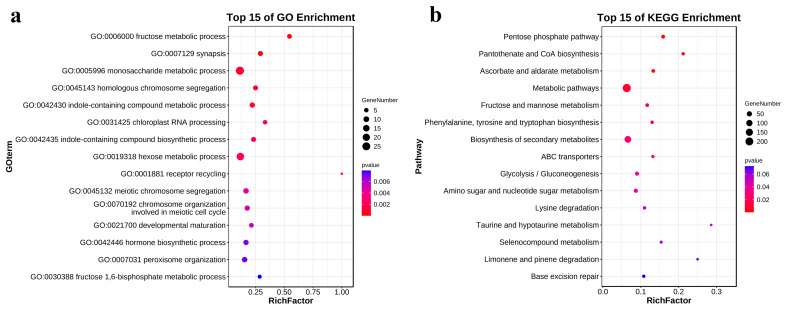
(**a**) Bubble plots of the top 15 GO items in the GO enrichment analysis of unique DEGs in the inbred line L318. (**b**) Bubble plots of the top 15 KEGG items in the GO enrichment analysis of unique DEGs in the inbred line L318.

The GO enrichment analysis of the unique genes in the salt-tolerant line revealed that certain DEGs were enriched in biological processes associated with sugar metabolism. These processes included the fructose metabolic process (GO:0006000), monosaccharide metabolic process (GO:0005996), and hormone biosynthetic process (GO:0042446) (Figure 4a). The pentose phosphate pathway (ko00030), fructose and mannose metabolism (ko00051), and glycolysis/gluconeogenesis (ko00010) were highly enriched in KEGG (Figure 4b).

### 3.4. Analysis of the Expression Trend of Salt-Tolerant Genes

**Figure 5:**
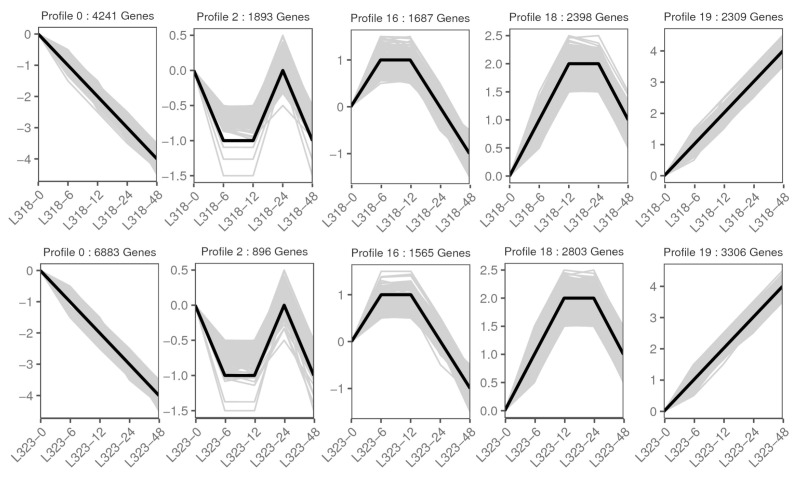
The expression trend of DEGs in the screened expression profile 0, 2, 16, 18, and 19.

A gene expression trend analysis could be utilized to identify DEGs that exhibited similar expression patterns at various time points. This analysis enabled the identification of gene sets that possessed specific biological characteristics. In this study, the DEGs of all the samples were analyzed using STEM, resulting in the identification of 20 gene profiles. Subsequently, for each inbred line, five significant sets of expression trends (*p* < 0.05) were determined. The expression trend of profile 0 demonstrated a continuous downregulation throughout the duration of the salt-stress treatment. Conversely, the genes in profile 19 exhibited a gradual upregulation over the treatment period. Both profile 16 and 18 displayed an “up-steady-down” trend. Profile 2 exhibited a “down-steady-up-down” trend (Figure 5).

Following that, a KEGG enrichment analysis was conducted on these profiles, and the specific results are presented in Table S3. Notably, the plant hormone signal transduction and MAPK signaling pathway–plant was found to be enriched to varying degrees in profile 16 and 18. These two expression trends were upregulated at 0–6 and 0–12 h after salt stress, indicating that these pathways occurred in the early stages of salt treatment and played a crucial role in the salt tolerance response in maize.

### 3.5. DEGs Involved in Plant Hormone Signal Transduction in Maize Root

**Figure 6:**
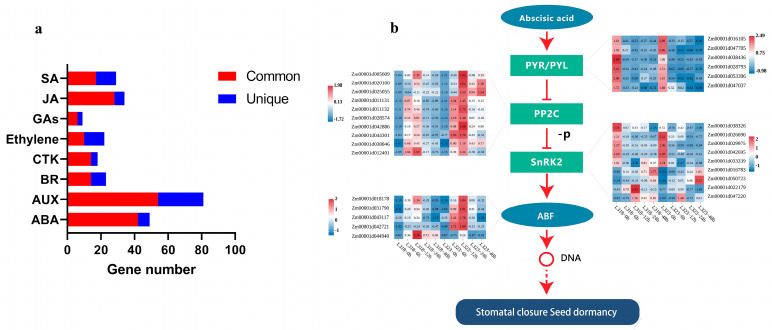
(**a**) The number of DEGs contained in various hormone pathways. (**b**) The abscisic acid signal pathway in maize roots.

In the KEGG analysis, a total of 219 and 240 DEGs were found to be associated with the plant hormone signal transduction pathway in L318 and L323, respectively (Table S4). This pathway encompassed auxin, cytokinin, gibberellin, abscisic acid, ethylene, jasmonic acid, brassinosteroid, and salicylic acid. Remarkably, there were a large number of common genes and abundant hormone components in each hormone signal pathway (Figure 6a and Table S5).

In the abscisic acid (ABA) signal pathway, a total of 49 genes exhibited differential expression. Among these genes, 42 were found to be present in both inbred lines, and 25 genes displayed a similar expression pattern. These genes were divided into protein phosphatase 2C (PP2C), sucrose non-fermenting-1-related protein kinase 2 (SnRK2), and ABA-responsive element binding factor (ABF). The expression of pyrabactin resistance/pyrabactin-resistance-like (PYR/PYL) were downregulated in both inbred lines, while PP2C demonstrated an initial upregulation followed by a subsequent downregulation. Notably, the expression levels of *Zm00001d020100* (*ZmPP2C11*) and *Zm00001d025055* (*ZmPP2C13*) exhibited significant differences between the two inbred lines. In SnRK2 and ABF, the differential expression of the same genes such as *Zm00001d050723* (*ZmSnRK2.6*) and *Zm00001d042721* (*ZmbZip37*) among the varieties was more pronounced (Figure 6b). Furthermore, the ABA hormone signal pathway was closely associated with the MAPK signaling pathway, which was induced by salt stress, cold stress, and osmotic stress. This close relationship between the ABA pathway and stress-induced MAPK signaling pathways warrants further attention and investigation.

### 3.6. DEGs Involved in the MAPK Signaling Pathway–Plant in Maize Roots

In L318 and L323, the MAPK signaling pathway–plants enriched 148 and 163 DEGs, with 125 of these genes shared by two inbred lines. Protein phosphorylation/dephosphorylation are fundamental mechanisms through which cells adapt to alterations in the external surroundings and modulate cell function. Protein phosphorylation catalyzed by protein kinase can amplify environmental signals step-by-step, and then regulate the cell physiological response. The MAPK cascade reaction pathway plays an important role in the transduction of environmental stress signals. During environmental stress, plants amplify these signals through a triple phosphorylation cascade mediated by MAPKKK-MAPKK-MAPK to induce tolerance responses.

**Figure 7:**
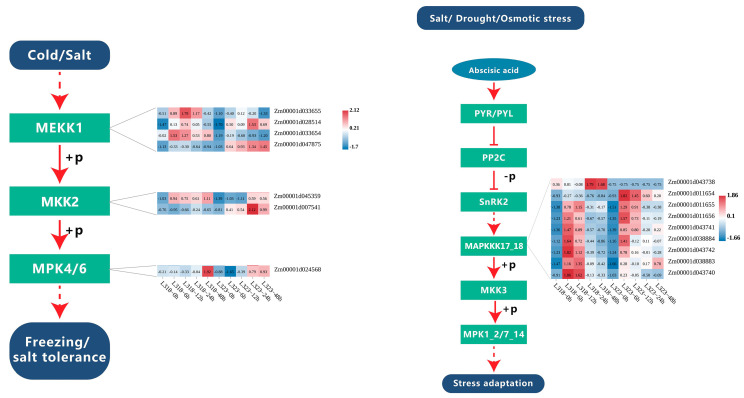
Two signaling pathways in MAPK signaling pathway–plant in maize roots.

The analysis of the KEGG pathway map revealed that salt stress affected two pathways, leading to salt tolerance through a series of signal transduction events. Among the DEGs involved in the MAPK signaling pathway, we focused on four components: MEKK1 (K13414), MKK2 (K20603), MPK4 (K20600), and MAPKKK17_18 (K20716). Under salt stress, MEKK1, MEKK2, and MPK4/6 were upregulated, with variations in their expressions observed among different lines. Notably, the upregulation of MEKK1 occurred in the early stage of stress, while the upregulation of MPK4 occurred in the late stage. In the case of MAPKKK17_18, *Zm00001d043738* was not expressed in the salt-sensitive line, and the expression level of *Zm00001d011654* in the salt-tolerant line was significantly lower than that in the sensitive line. The other genes showed upregulation and reached their peak expression levels at 6 and 12 h (Figure 7).

### 3.7. Changes in Differentially Expressed TFs under Salt Stress

Transcription factors (TFs) are crucial regulators of gene expression and play a pivotal role in governing plant growth, development, and response to stress. Through a comparative analysis of gene expression profiles in the roots of two inbred maize lines subjected to NaCl stress at various time points, a total of 1326 TFs from 51 TF families were identified. Notably, the type-B Arabidopsis response regulator (ARR-B), APETALA2/Ethylene-responsive element binding proteins (AP2-EREBP), and the basic helix–loop–helix (bHLH), WRKY, NAC, and basic leucine zipper (bZIP) families exhibited a significant number of DEGs associated with salt tolerance in maize. Moreover, a subset of 150 DEGs consistently displayed differential expression throughout the entire duration of salt treatment (Figure S1).

### 3.8. Validation of Candidate Gene Expression

**Figure 8:**
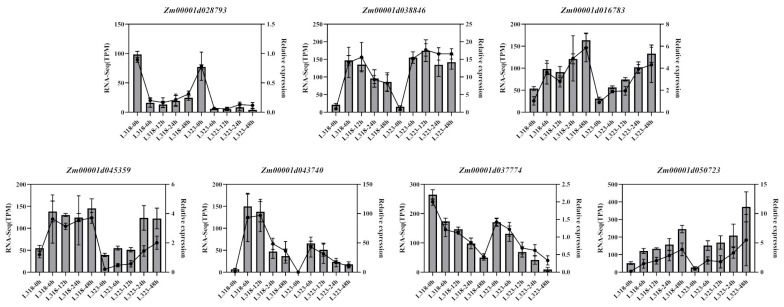
qPCR validation of seven gene expression levels, with a bar chart representing RNA Seq expression levels (TPM) and a line chart representing relative expression levels.

To validate the accuracy of the RNA-Seq data, seven genes associated with ABA signal transduction and the MAPK signaling pathway were chosen at random for qRT-PCR. As shown in Figure 8, the RT-PCR results of these genes were consistent with the expression patterns of the RNA-Seq data. Genes significantly upregulated in RNA-Seq data also exhibited a significant upregulation in qPCR, and vice versa. These results also confirmed the reliability of RNA-Seq data.

## 4. Discussion

Salt stress can lead to the yellowing of plant leaves, decreased biomass, delayed plant development, reduced panicles, and lower thousand-grain weight, ultimately affecting the harvest index and grain yield [29,30]. Maize exhibits a certain degree of susceptibility to salt stress, and salt tolerance varies among different genotypes. Consequently, we selected two maize inbred lines for further evaluation of their salt tolerance. The results indicated that salt stress hindered the growth of seedlings in both genotypes. However, L318 exhibited greater tolerance compared with L323. The root system is responsible for water and nutrient acquisition and is involved in the plant’s response to various abiotic stresses [31]. Transcriptome analysis can provide insights into the plant’s response to salt stress by examining overall patterns of gene expression [32]. However, many studies on the transcriptome of salt-tolerant maize have only collected expression levels at the beginning and end of salt stress [33,34,35,36]. Therefore, we chose to investigate the dynamic global transcriptome on the root systems of seedlings at five different time points after salt treatment and compared transcriptome data from different genotypes to examine the similarities and differences in salt tolerance mechanisms of different germplasms.

Plant hormones are small molecules that regulate plant growth and development, as well as signaling molecules within the plants. They can facilitate the transmission of biological signals and govern the regulation of growth and development processes [37]. It is worth noting that we observed a significant differential expression of genes encoding hormone signals in the early stages of stress, and these DEGs were abundant in the abscisic acid signal pathways. Previous studies have shown that ABA is the primary hormone involved in the plant’s response to abiotic stresses [38]. Under salt stress, ABA activates the expression of downstream stress genes, regulating ion balance and metabolic changes [3,39]. In this study, we identified some DEGs that were significantly enriched in the ABA signal pathway and found the connection between the ABA signal pathway and the MAPK signaling pathway. Salt stress may have induced the change in the PYR/PYL expression levels in the ABA signal pathway, thus relieving the inhibition of SnRK2 with PP2C and indirectly activating the MAPK signaling pathway. This gradually amplified environmental stress signals and ultimately promoted a plant stress response. Additionally, we also discovered several ABFs annotated as bZIP transcription factors, including *Zm00001d018178*, which has been demonstrated to contribute to the development and resilience of maize roots in the face of abiotic stresses. The overexpression of this gene can improve the survival of plants subjected to severe abiotic stresses [40]. In addition to the ABA signal pathway, we also detected components of several other hormone signal pathways (Table S5). Certain hormones like auxin were found to enhance the adaptability of plants to adversity, while the exogenous application of ethylene can improve the sensitivity of rice seedlings to salt [41]. Taken together, the induction of hormone signal expression by stressful environments and the enhancement or decrease of salt tolerance mediated by hormone signals are not single or unrelated. Instead, multiple hormone signals and their components have a cross-talk relationship in the regulation of abiotic stresses. Consequently, a detailed investigation of the response mechanism of this extensive and intricate regulatory network to abiotic stresses is warranted.

Salt tolerance in plants is achieved through the coordination of cellular processes, molecular interactions, and metabolic pathways [42]. Plants have evolved a network of signaling pathways to cope with various types of abiotic stresses, with the MAPK signaling pathway playing a crucial role in effectively responding to external stimuli [43]. The MAPK pathway consists of intracellular signaling factors that transmit external signals to the cell through a three-layer protein kinase cascade, involving MAPKKKs, MAPKKs, and MAPKs [44]. The activation of MAPK and its downstream genes can influence plant stress resistance [45]. In the KEGG pathway map, two MAPK signaling pathways were relevant to this study. One of these pathways is associated with the ABA signal pathway, which is located downstream. The overexpression of PeMKK2a in poplar was shown to enhance the scavenging of reactive oxygen species under salt stress, resulting in improved salt tolerance in poplar trees [46]. In our study, we observed the activation of specific key components of the MAPK signaling pathway during salt stress, with noticeable differences in expression levels between the two germplasms. *Zm00001d043738* exhibited differential expression in L318 but not in L323. *Zm00001d043741* initially showed upregulation followed by downregulation in both inbred lines, with significantly higher peak expression in L318 compared with L323. Previous studies have indicated that NPK1, isolated from tobacco, positively regulated drought tolerance, and transgenic maize and rice-expressing NPK1 exhibited higher yields under drought conditions. *ZmMAPKKK18* (*Zm00001d043741*), which shares high homology with NPK1, is upregulated in leaves, stems, and roots, suggesting its potential involvement in regulating the response to drought stress [47]. The expression of *Zm00001d043742* peaked at 6 h in both lines, with levels over 50 times higher than those at 0 h. Although the orthologous gene *OsMAPKKK63* (*Os01g0699100*) has been found to be involved in the response to high salt stress and the regulation of seed dormancy in *Oryza sativa japonica*, its presence in maize has not been reported. These findings indicated that the MAPKKK gene family actively responds to salt stress.

Transcription factors are widely recognized for their significant involvement in plant responses to salt stress. Our research findings indicated that, under salt-stress conditions, the shared transcription factors in the two inbred lines were primarily concentrated within six families: ARR-B, AP2-EREBP, bHLH, WRKY, NAC, and bZIP. Previous studies have demonstrated that various transcription factors, including WRKY, bZIP, and bHLH, were induced by salt stress and contributed to the plant’s ability to respond to drought and chilling stress [48,49,50]. Among these transcription factors, *Zm00001d033957* (*ZmbHLH103*) was shown to play a crucial role in enhancing maize’s tolerance to drought [51]. Additionally, the overexpression of *Zm00001d013003* (*ZmNAC55*) in Arabidopsis was found to improve the drought tolerance of seedlings [52].

## 5. Conclusions

In conclusion, this study conducted a comparative transcriptome analysis of salt tolerance in maize based on root transcriptome datasets of two inbred lines and discovered that the MAPK signaling pathway and plant hormone signal pathway played a role in coordinating the salt tolerance response of maize roots. The KEGG pathway map revealed a relationship between the ABA signal pathway and the MAPK signaling pathway. Furthermore, the KEGG analysis of specific genes in the salt-tolerant line L318 indicated enrichment in the pentose phosphate pathway and the glycolysis/gluconeogenesis pathway, which may have contributed to its stronger salt tolerance. Our research indicated that there were similarities and differences in the responses of CML115 and GEMS58 to salt stress, and the findings provide a reference for studying the salt tolerance mechanisms of maize.

## Data Availability

The data presented in this research are available on request from the corresponding author. Data is not publicly available due to privacy.

## Supplementary Materials

The following are available online at https://www.mdpi.com/article/10.3390/metabo13111155/s1. Figure S1: All DEG encoded transcription factors (TFs) during the salt treatment stage; Table S1: All reads information; Table S2: All alignment statistics; Table S3: Trend KEGG enrichment; Table S4: DEGs involved in signal transduction; Table S5: DEGs involved in plant hormone signal transduction; Table S6: Primers used for qRT-PCR.

## References

[1] Ismail A.M., Horie T. (2017). Genomics, Physiology, and Molecular Breeding Approaches for Improving Salt Tolerance. Annu. Rev. Plant Biol..

[2] Jiao Y., Zhao H., Ren L., Song W., Zeng B., Guo J., Wang B., Liu Z., Chen J., Li W. (2012). Genome-wide genetic changes during modern breeding of maize. Nat. Genet..

[3] van Zelm E., Zhang Y., Testerink C. (2020). Salt Tolerance Mechanisms of Plants. Annu. Rev. Plant Biol..

[4] Yu Z., Duan X., Luo L., Dai S., Ding Z., Xia G. (2020). How Plant Hormones Mediate Salt Stress Responses. Trends Plant Sci..

[5] Hake S., Ross-Ibarra J. (2015). Genetic, evolutionary and plant breeding insights from the domestication of maize. Elife.

[6] Munns R., James R.A., Xu B., Athman A., Conn S.J., Jordans C., Byrt C.S., Hare R.A., Tyerman S.D., Tester M. (2012). Wheat grain yield on saline soils is improved by an ancestral Na⁺ transporter gene. Nat. Biotechnol..

[7] Sandhu D., Pudussery M.V., Kumar R., Pallete A., Markley P., Bridges W.C., Sekhon R.S. (2020). Characterization of natural genetic variation identifies multiple genes involved in salt tolerance in maize. Funct. Integr. Genom..

[8] Deinlein U., Stephan A.B., Horie T., Luo W., Xu G., Schroeder J.I. (2014). Plant salt-tolerance mechanisms. Trends Plant Sci..

[9] Munns R. (2005). Genes and salt tolerance: Bringing them together. New Phytol..

[10] Ahmad P., Abdel Latef A.A., Hashem A., Abd Allah E.F., Gucel S., Tran L.S. (2016). Nitric Oxide Mitigates Salt Stress by Regulating Levels of Osmolytes and Antioxidant Enzymes in Chickpea. Front. Plant Sci..

[11] Gill S.S., Tuteja N. (2010). Reactive oxygen species and antioxidant machinery in abiotic stress tolerance in crop plants. Plant Physiol. Biochem..

[12] Chen J., Zhang J., Hu J., Xiong W., Du C., Lu M. (2017). Integrated regulatory network reveals the early salt tolerance mechanism of Populus euphratica. Sci. Rep..

[13] Munns R., Tester M. (2008). Mechanisms of salinity tolerance. Annu. Rev. Plant Biol..

[14] Liang W., Ma X., Wan P., Liu L. (2018). Plant salt-tolerance mechanism: A review. Biochem. Biophys. Res. Commun..

[15] Yin P., Liang X., Zhao H., Xu Z., Chen L., Yang X., Qin F., Zhang J., Jiang C. (2023). Cytokinin signaling promotes salt tolerance by modulating shoot chloride exclusion in maize. Mol. Plant.

[16] Fu J., Zhu C., Wang C., Liu L., Shen Q., Xu D., Wang Q. (2021). Maize transcription factor ZmEREB20 enhanced salt tolerance in transgenic Arabidopsis. Plant Physiol. Biochem..

[17] Bo C., Chen H., Luo G., Li W., Zhang X., Ma Q., Cheng B., Cai R. (2020). Maize WRKY114 gene negatively regulates salt-stress tolerance in transgenic rice. Plant Cell Rep..

[18] Liu C., Zhao Y., Zhao X., Wang J., Gu M., Yuan Z. (2020). Transcriptomic Profiling of Pomegranate Provides Insights into Salt Tolerance. Agronomy.

[19] Cui D., Wu D., Somarathna Y., Xu C., Li S., Li P., Zhang H., Chen H., Zhao L. (2015). QTL mapping for salt tolerance based on snp markers at the seedling stage in maize (*Zea mays* L.). Euphytica.

[20] Zhang M., Cao Y., Wang Z., Wang Z.Q., Shi J., Liang X., Song W., Chen Q., Lai J., Jiang C. (2018). A retrotransposon in an HKT1 family sodium transporter causes variation of leaf Na(+) exclusion and salt tolerance in maize. New Phytol..

[21] Sharmin R.A., Bhuiyan M.R., Lv W., Yu Z., Chang F., Kong J., Bhat J.A., Zhao T. (2020). RNA-Seq based transcriptomic analysis revealed genes associated with seed-flooding tolerance in wild soybean (*Glycine soja* Sieb. & Zucc.). Environ. Exp. Bot..

[22] Wang H., Miyazaki S., Kawai K., Deyholos M., Galbraith D.W., Bohnert H.J. (2003). Temporal progression of gene expression responses to salt shock in maize roots. Plant Mol. Biol..

[23] Chen C., Shang X., Sun M., Tang S., Khan A., Zhang D., Yan H., Jiang Y., Yu F., Wu Y. (2022). Comparative Transcriptome Analysis of Two Sweet Sorghum Genotypes with Different Salt Tolerance Abilities to Reveal the Mechanism of Salt Tolerance. Int. J. Mol. Sci..

[24] Zhang X., Liu J., Huang Y., Wu H., Hu X., Cheng B., Ma Q., Zhao Y. (2022). Comparative Transcriptomics Reveals the Molecular Mechanism of the Parental Lines of Maize Hybrid An’nong876 in Response to Salt Stress. Int. J. Mol. Sci..

[25] Love M.I., Huber W., Anders S. (2014). Moderated estimation of fold change and dispersion for RNA-seq data with DESeq2. Genome Biol..

[26] Robinson M.D., McCarthy D.J., Smyth G.K. (2010). edgeR: A Bioconductor package for differential expression analysis of digital gene expression data. Bioinformatics.

[27] Kanehisa M., Goto S. (2000). KEGG: Kyoto encyclopedia of genes and genomes. Nucleic Acids Res..

[28] Livak K.J., Schmittgen T.D. (2001). Analysis of relative gene expression data using real-time quantitative PCR and the 2(-Delta Delta C(T)) Method. Methods.

[29] Razzaq A., Ali A., Safdar L.B., Zafar M.M., Rui Y., Shakeel A., Shaukat A., Ashraf M., Gong W., Yuan Y. (2020). Salt stress induces physiochemical alterations in rice grain composition and quality. J. Food Sci..

[30] Hakim M.A., Juraimi A.S., Hanafi M.M., Ismail M.R., Selamat A., Rafii M.Y., Latif M.A. (2014). Biochemical and anatomical changes and yield reduction in rice (*Oryza sativa* L.) under varied salinity regimes. Biomed. Res. Int..

[31] Saini S., Sharma I., Kaur N., Pati P.K. (2013). Auxin: A master regulator in plant root development. Plant Cell Rep..

[32] Chinnusamy V., Jagendorf A., Zhu J.-K. (2005). Understanding and Improving Salt Tolerance in Plants. Crop Sci..

[33] Fan X., Jiang H., Meng L., Chen J. (2021). Gene Mapping, Cloning and Association Analysis for Salt Tolerance in Rice. Int. J. Mol. Sci..

[34] Buchanan C.D., Lim S., Salzman R.A., Kagiampakis I., Morishige D.T., Weers B.D., Klein R.R., Pratt L.H., Cordonnier-Pratt M.M., Klein P.E. (2005). Sorghum bicolor’s transcriptome response to dehydration, high salinity and ABA. Plant Mol. Biol..

[35] Sui N., Yang Z., Liu M., Wang B. (2015). Identification and transcriptomic profiling of genes involved in increasing sugar content during salt stress in sweet sorghum leaves. BMC Genom..

[36] Ukwatta J., Pabuayon I.C.M., Park J., Chen J., Chai X., Zhang H., Zhu J.K., Xin Z., Shi H. (2021). Comparative physiological and transcriptomic analysis reveals salinity tolerance mechanisms in *Sorghum bicolor* (L.) Moench. Planta.

[37] Seif El-Yazal S.A., Seif El-Yazal M.A., Dwidar E.F., Rady M.M. (2015). Phytohormone crosstalk research: Cytokinin and its crosstalk with other phytohormones. Curr. Protein Pept. Sci..

[38] Finkelstein R. (2013). Abscisic Acid synthesis and response. Arab. Book.

[39] Belda-Palazón B., Adamo M., Valerio C., Ferreira L.J., Confraria A., Reis-Barata D., Rodrigues A., Meyer C., Rodriguez P.L., Baena-González E. (2020). A dual function of SnRK2 kinases in the regulation of SnRK1 and plant growth. Nat. Plants.

[40] Lv B., Yan Z., Tian H., Zhang X., Ding Z. (2019). Local Auxin Biosynthesis Mediates Plant Growth and Development. Trends Plant Sci..

[41] Liu C., Mao B., Yuan D., Chu C., Duan M. (2022). Salt tolerance in rice: Physiological responses and molecular mechanisms. Crop J..

[42] Amirbakhtiar N., Ismaili A., Ghaffari M.R., Mirdar Mansuri R., Sanjari S., Shobbar Z.S. (2021). Transcriptome analysis of bread wheat leaves in response to salt stress. PLoS ONE.

[43] Yao Y., Zhao H., Sun L., Wu W., Li C., Wu Q. (2022). Genome-wide identification of MAPK gene family members in Fagopyrum tataricum and their expression during development and stress responses. BMC Genom..

[44] Coulthard L.R., White D.E., Jones D.L., McDermott M.F., Burchill S.A. (2009). p38(MAPK): Stress responses from molecular mechanisms to therapeutics. Trends Mol. Med..

[45] Wei L., Feng L., Liu Y., Liao W. (2022). Mitogen-Activated Protein Kinase Is Involved in Salt Stress Response in Tomato (*Solanum lycopersicum*) Seedlings. Int. J. Mol. Sci..

[46] Wang J., Sun Z., Chen C., Xu M. (2022). The MKK2a Gene Involved in the MAPK Signaling Cascades Enhances Populus Salt Tolerance. Int. J. Mol. Sci..

[47] Liu Y., Zhou M., Gao Z., Ren W., Yang F., He H., Zhao J. (2015). RNA-Seq Analysis Reveals MAPKKK Family Members Related to Drought Tolerance in Maize. PLoS ONE.

[48] Cheng Z., Luan Y., Meng J., Sun J., Tao J., Zhao D. (2021). WRKY Transcription Factor Response to High-Temperature Stress. Plants.

[49] Zhang B., Feng C., Chen L., Li B., Zhang X., Yang X. (2022). Identification and Functional Analysis of bZIP Genes in Cotton Response to Drought Stress. Int. J. Mol. Sci..

[50] Jin R., Kim H.S., Yu T., Zhang A., Yang Y., Liu M., Yu W., Zhao P., Zhang Q., Cao Q. (2021). Identification and function analysis of bHLH genes in response to cold stress in sweetpotato. Plant Physiol. Biochem..

[51] Waititu J.K., Zhang X., Chen T., Zhang C., Zhao Y., Wang H. (2021). Transcriptome Analysis of Tolerant and Susceptible Maize Genotypes Reveals Novel Insights about the Molecular Mechanisms Underlying Drought Responses in Leaves. Int. J. Mol. Sci..

[52] Mao H., Yu L., Han R., Li Z., Liu H. (2016). ZmNAC55, a maize stress-responsive NAC transcription factor, confers drought resistance in transgenic Arabidopsis. Plant Physiol. Biochem..

