# Supplementary figures and images for: Comparative Transcriptome Analysis Reveals the Underlying Response Mechanism to Salt Stress in Maize Seedling Roots
Source: Metabolites. 2023 Nov 16;13(11):1155. doi: 10.3390/metabo13111155 (PMC10673138; doi:10.3390/metabo13111155)

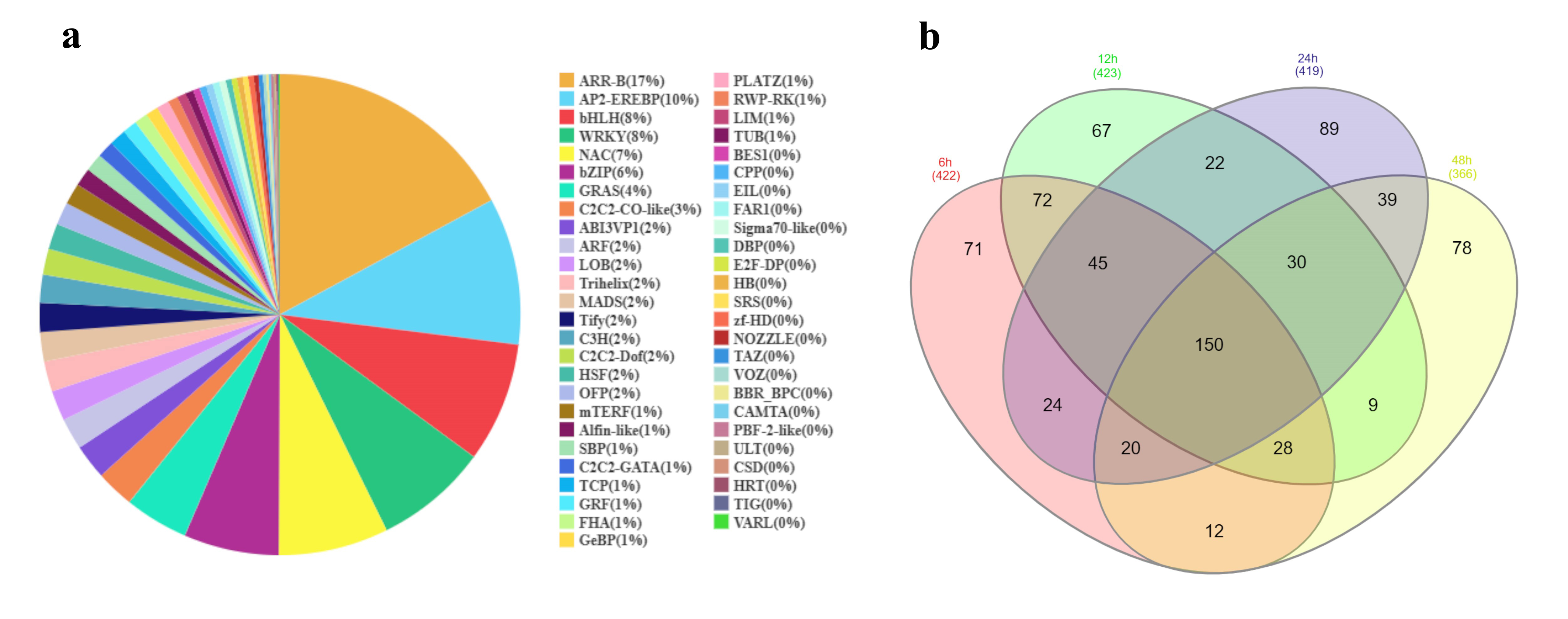

Supplement: Supplementary file 1 [file metabolites-13-01155-s001.zip › Figure S1.jpg]
